# Supplementary material for: The Crohn’s disease polymorphism, ATG16L1 T300A, alters the gut microbiota and enhances the local Th1/Th17 response
Source: eLife. 2019 Jan 22;8:e39982. doi: 10.7554/eLife.39982 (PMC6342529; doi:10.7554/eLife.39982)
Supplement: Supplementary file 1. — Mouse genotype, age (wks), sex, cage, experiment description and figure are listed above. Mice housed together have the same cage number. [file elife-39982-supp1.pptx]

## Slide 1
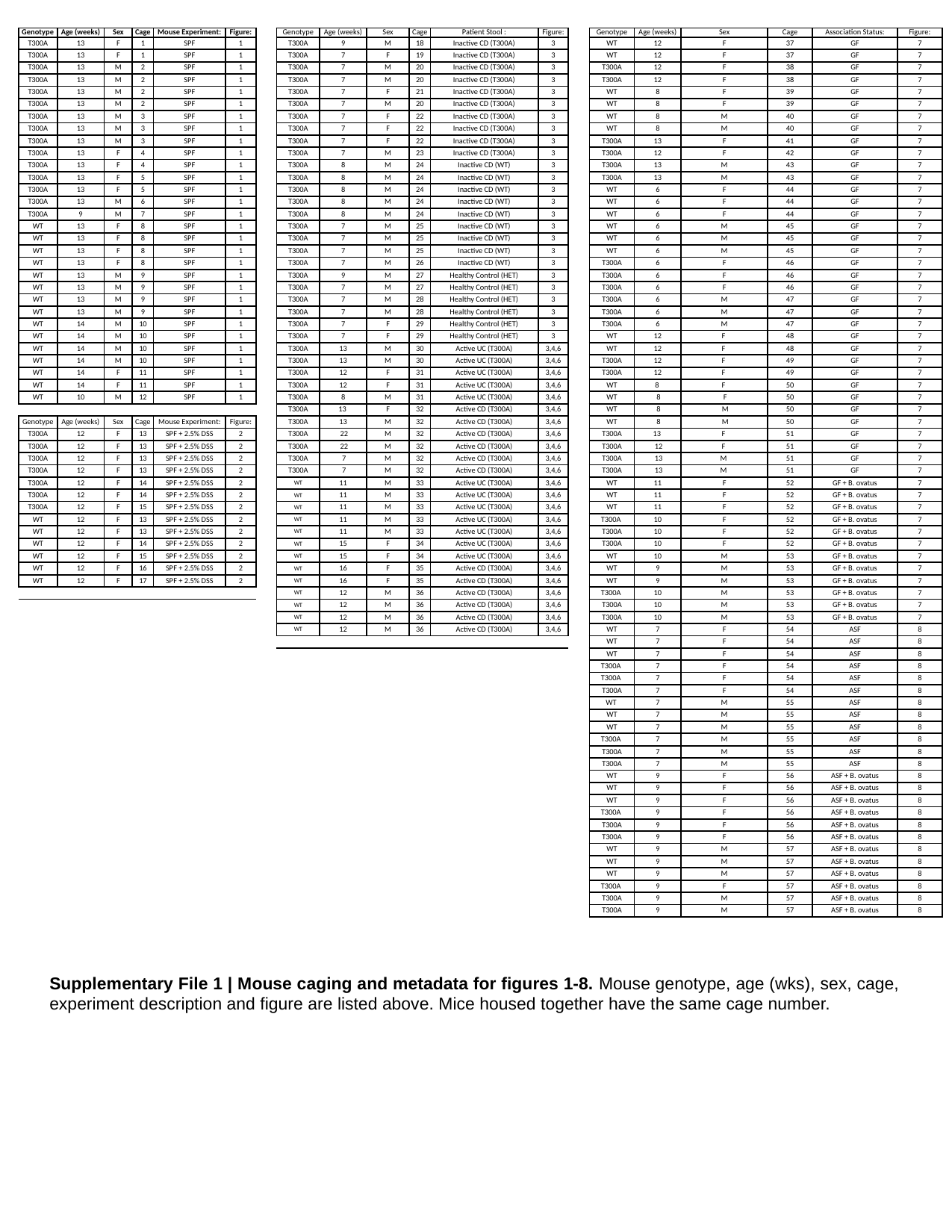

| Genotype | Age (weeks) | Sex | Cage | Mouse Experiment: | Figure: | | Genotype | Age (weeks) | Sex | Cage | Patient Stool : | Figure: | | Genotype | Age (weeks) | Sex | Cage | Association Status: | Figure: |
| --- | --- | --- | --- | --- | --- | --- | --- | --- | --- | --- | --- | --- | --- | --- | --- | --- | --- | --- | --- |
| T300A | 13 | F | 1 | SPF | 1 | | T300A | 9 | M | 18 | Inactive CD (T300A) | 3 | | WT | 12 | F | 37 | GF | 7 |
| T300A | 13 | F | 1 | SPF | 1 | | T300A | 7 | F | 19 | Inactive CD (T300A) | 3 | | WT | 12 | F | 37 | GF | 7 |
| T300A | 13 | M | 2 | SPF | 1 | | T300A | 7 | M | 20 | Inactive CD (T300A) | 3 | | T300A | 12 | F | 38 | GF | 7 |
| T300A | 13 | M | 2 | SPF | 1 | | T300A | 7 | M | 20 | Inactive CD (T300A) | 3 | | T300A | 12 | F | 38 | GF | 7 |
| T300A | 13 | M | 2 | SPF | 1 | | T300A | 7 | F | 21 | Inactive CD (T300A) | 3 | | WT | 8 | F | 39 | GF | 7 |
| T300A | 13 | M | 2 | SPF | 1 | | T300A | 7 | M | 20 | Inactive CD (T300A) | 3 | | WT | 8 | F | 39 | GF | 7 |
| T300A | 13 | M | 3 | SPF | 1 | | T300A | 7 | F | 22 | Inactive CD (T300A) | 3 | | WT | 8 | M | 40 | GF | 7 |
| T300A | 13 | M | 3 | SPF | 1 | | T300A | 7 | F | 22 | Inactive CD (T300A) | 3 | | WT | 8 | M | 40 | GF | 7 |
| T300A | 13 | M | 3 | SPF | 1 | | T300A | 7 | F | 22 | Inactive CD (T300A) | 3 | | T300A | 13 | F | 41 | GF | 7 |
| T300A | 13 | F | 4 | SPF | 1 | | T300A | 7 | M | 23 | Inactive CD (T300A) | 3 | | T300A | 12 | F | 42 | GF | 7 |
| T300A | 13 | F | 4 | SPF | 1 | | T300A | 8 | M | 24 | Inactive CD (WT) | 3 | | T300A | 13 | M | 43 | GF | 7 |
| T300A | 13 | F | 5 | SPF | 1 | | T300A | 8 | M | 24 | Inactive CD (WT) | 3 | | T300A | 13 | M | 43 | GF | 7 |
| T300A | 13 | F | 5 | SPF | 1 | | T300A | 8 | M | 24 | Inactive CD (WT) | 3 | | WT | 6 | F | 44 | GF | 7 |
| T300A | 13 | M | 6 | SPF | 1 | | T300A | 8 | M | 24 | Inactive CD (WT) | 3 | | WT | 6 | F | 44 | GF | 7 |
| T300A | 9 | M | 7 | SPF | 1 | | T300A | 8 | M | 24 | Inactive CD (WT) | 3 | | WT | 6 | F | 44 | GF | 7 |
| WT | 13 | F | 8 | SPF | 1 | | T300A | 7 | M | 25 | Inactive CD (WT) | 3 | | WT | 6 | M | 45 | GF | 7 |
| WT | 13 | F | 8 | SPF | 1 | | T300A | 7 | M | 25 | Inactive CD (WT) | 3 | | WT | 6 | M | 45 | GF | 7 |
| WT | 13 | F | 8 | SPF | 1 | | T300A | 7 | M | 25 | Inactive CD (WT) | 3 | | WT | 6 | M | 45 | GF | 7 |
| WT | 13 | F | 8 | SPF | 1 | | T300A | 7 | M | 26 | Inactive CD (WT) | 3 | | T300A | 6 | F | 46 | GF | 7 |
| WT | 13 | M | 9 | SPF | 1 | | T300A | 9 | M | 27 | Healthy Control (HET) | 3 | | T300A | 6 | F | 46 | GF | 7 |
| WT | 13 | M | 9 | SPF | 1 | | T300A | 7 | M | 27 | Healthy Control (HET) | 3 | | T300A | 6 | F | 46 | GF | 7 |
| WT | 13 | M | 9 | SPF | 1 | | T300A | 7 | M | 28 | Healthy Control (HET) | 3 | | T300A | 6 | M | 47 | GF | 7 |
| WT | 13 | M | 9 | SPF | 1 | | T300A | 7 | M | 28 | Healthy Control (HET) | 3 | | T300A | 6 | M | 47 | GF | 7 |
| WT | 14 | M | 10 | SPF | 1 | | T300A | 7 | F | 29 | Healthy Control (HET) | 3 | | T300A | 6 | M | 47 | GF | 7 |
| WT | 14 | M | 10 | SPF | 1 | | T300A | 7 | F | 29 | Healthy Control (HET) | 3 | | WT | 12 | F | 48 | GF | 7 |
| WT | 14 | M | 10 | SPF | 1 | | T300A | 13 | M | 30 | Active UC (T300A) | 3,4,6 | | WT | 12 | F | 48 | GF | 7 |
| WT | 14 | M | 10 | SPF | 1 | | T300A | 13 | M | 30 | Active UC (T300A) | 3,4,6 | | T300A | 12 | F | 49 | GF | 7 |
| WT | 14 | F | 11 | SPF | 1 | | T300A | 12 | F | 31 | Active UC (T300A) | 3,4,6 | | T300A | 12 | F | 49 | GF | 7 |
| WT | 14 | F | 11 | SPF | 1 | | T300A | 12 | F | 31 | Active UC (T300A) | 3,4,6 | | WT | 8 | F | 50 | GF | 7 |
| WT | 10 | M | 12 | SPF | 1 | | T300A | 8 | M | 31 | Active UC (T300A) | 3,4,6 | | WT | 8 | F | 50 | GF | 7 |
| | | | | | | | T300A | 13 | F | 32 | Active CD (T300A) | 3,4,6 | | WT | 8 | M | 50 | GF | 7 |
| Genotype | Age (weeks) | Sex | Cage | Mouse Experiment: | Figure: | | T300A | 13 | M | 32 | Active CD (T300A) | 3,4,6 | | WT | 8 | M | 50 | GF | 7 |
| T300A | 12 | F | 13 | SPF + 2.5% DSS | 2 | | T300A | 22 | M | 32 | Active CD (T300A) | 3,4,6 | | T300A | 13 | F | 51 | GF | 7 |
| T300A | 12 | F | 13 | SPF + 2.5% DSS | 2 | | T300A | 22 | M | 32 | Active CD (T300A) | 3,4,6 | | T300A | 12 | F | 51 | GF | 7 |
| T300A | 12 | F | 13 | SPF + 2.5% DSS | 2 | | T300A | 7 | M | 32 | Active CD (T300A) | 3,4,6 | | T300A | 13 | M | 51 | GF | 7 |
| T300A | 12 | F | 13 | SPF + 2.5% DSS | 2 | | T300A | 7 | M | 32 | Active CD (T300A) | 3,4,6 | | T300A | 13 | M | 51 | GF | 7 |
| T300A | 12 | F | 14 | SPF + 2.5% DSS | 2 | | WT | 11 | M | 33 | Active UC (T300A) | 3,4,6 | | WT | 11 | F | 52 | GF + B. ovatus | 7 |
| T300A | 12 | F | 14 | SPF + 2.5% DSS | 2 | | WT | 11 | M | 33 | Active UC (T300A) | 3,4,6 | | WT | 11 | F | 52 | GF + B. ovatus | 7 |
| T300A | 12 | F | 15 | SPF + 2.5% DSS | 2 | | WT | 11 | M | 33 | Active UC (T300A) | 3,4,6 | | WT | 11 | F | 52 | GF + B. ovatus | 7 |
| WT | 12 | F | 13 | SPF + 2.5% DSS | 2 | | WT | 11 | M | 33 | Active UC (T300A) | 3,4,6 | | T300A | 10 | F | 52 | GF + B. ovatus | 7 |
| WT | 12 | F | 13 | SPF + 2.5% DSS | 2 | | WT | 11 | M | 33 | Active UC (T300A) | 3,4,6 | | T300A | 10 | F | 52 | GF + B. ovatus | 7 |
| WT | 12 | F | 14 | SPF + 2.5% DSS | 2 | | WT | 15 | F | 34 | Active UC (T300A) | 3,4,6 | | T300A | 10 | F | 52 | GF + B. ovatus | 7 |
| WT | 12 | F | 15 | SPF + 2.5% DSS | 2 | | WT | 15 | F | 34 | Active UC (T300A) | 3,4,6 | | WT | 10 | M | 53 | GF + B. ovatus | 7 |
| WT | 12 | F | 16 | SPF + 2.5% DSS | 2 | | WT | 16 | F | 35 | Active CD (T300A) | 3,4,6 | | WT | 9 | M | 53 | GF + B. ovatus | 7 |
| WT | 12 | F | 17 | SPF + 2.5% DSS | 2 | | WT | 16 | F | 35 | Active CD (T300A) | 3,4,6 | | WT | 9 | M | 53 | GF + B. ovatus | 7 |
| | | | | | | | WT | 12 | M | 36 | Active CD (T300A) | 3,4,6 | | T300A | 10 | M | 53 | GF + B. ovatus | 7 |
| | | | | | | | WT | 12 | M | 36 | Active CD (T300A) | 3,4,6 | | T300A | 10 | M | 53 | GF + B. ovatus | 7 |
| | | | | | | | WT | 12 | M | 36 | Active CD (T300A) | 3,4,6 | | T300A | 10 | M | 53 | GF + B. ovatus | 7 |
| | | | | | | | WT | 12 | M | 36 | Active CD (T300A) | 3,4,6 | | WT | 7 | F | 54 | ASF | 8 |
| | | | | | | | | | | | | | | WT | 7 | F | 54 | ASF | 8 |
| | | | | | | | | | | | | | | WT | 7 | F | 54 | ASF | 8 |
| | | | | | | | | | | | | | | T300A | 7 | F | 54 | ASF | 8 |
| | | | | | | | | | | | | | | T300A | 7 | F | 54 | ASF | 8 |
| | | | | | | | | | | | | | | T300A | 7 | F | 54 | ASF | 8 |
| | | | | | | | | | | | | | | WT | 7 | M | 55 | ASF | 8 |
| | | | | | | | | | | | | | | WT | 7 | M | 55 | ASF | 8 |
| | | | | | | | | | | | | | | WT | 7 | M | 55 | ASF | 8 |
| | | | | | | | | | | | | | | T300A | 7 | M | 55 | ASF | 8 |
| | | | | | | | | | | | | | | T300A | 7 | M | 55 | ASF | 8 |
| | | | | | | | | | | | | | | T300A | 7 | M | 55 | ASF | 8 |
| | | | | | | | | | | | | | | WT | 9 | F | 56 | ASF + B. ovatus | 8 |
| | | | | | | | | | | | | | | WT | 9 | F | 56 | ASF + B. ovatus | 8 |
| | | | | | | | | | | | | | | WT | 9 | F | 56 | ASF + B. ovatus | 8 |
| | | | | | | | | | | | | | | T300A | 9 | F | 56 | ASF + B. ovatus | 8 |
| | | | | | | | | | | | | | | T300A | 9 | F | 56 | ASF + B. ovatus | 8 |
| | | | | | | | | | | | | | | T300A | 9 | F | 56 | ASF + B. ovatus | 8 |
| | | | | | | | | | | | | | | WT | 9 | M | 57 | ASF + B. ovatus | 8 |
| | | | | | | | | | | | | | | WT | 9 | M | 57 | ASF + B. ovatus | 8 |
| | | | | | | | | | | | | | | WT | 9 | M | 57 | ASF + B. ovatus | 8 |
| | | | | | | | | | | | | | | T300A | 9 | F | 57 | ASF + B. ovatus | 8 |
| | | | | | | | | | | | | | | T300A | 9 | M | 57 | ASF + B. ovatus | 8 |
| | | | | | | | | | | | | | | T300A | 9 | M | 57 | ASF + B. ovatus | 8 |
Supplementary File 1 | Mouse caging and metadata for figures 1-8. Mouse genotype, age (wks), sex, cage, experiment description and figure are listed above. Mice housed together have the same cage number.
